# Umami Enhancing Properties of Enzymatically Hydrolyzed Mycelium of *Flammulina velutipes* Cultured on Potato Pulp

**DOI:** 10.1002/fsn3.70128

**Published:** 2025-03-31

**Authors:** Katharina Happel, Lea Zeller, Andreas Klaus Hammer, Holger Zorn

**Affiliations:** ^1^ Fraunhofer Institute for Molecular Biology and Applied Ecology IME Giessen Germany; ^2^ Institute of Food Chemistry and Food Biotechnology Justus‐ Liebig University Giessen Germany

**Keywords:** *Flammulina filiformis*, *Flammulina velutipes*, potato pulp, protein hydrolysis, taste enhancer, umami

## Abstract

The aim of this study was to hydrolyze cultivated fungal mycelium and to evaluate the effect on its taste. Potato pulp, a by‐product of the potato starch industry, was therefore successfully utilized as a substrate for submerged cultivation of *Flammulina velutipes*, yielding a product with an estimated fungal content of 83% ± 3%. The fermentation increased the protein content from 5.3 ± 0.4 g/100 g DM to 13.9 ± 0.1 g/100 g DM with a biological protein value of 86. The fermentate was enzymatically hydrolyzed by Corolase APC‐peptidase. After optimization of the hydrolysis conditions, a degree of hydrolysis (DH) of 75.1% ± 1.0% was achieved. The protein hydrolysis increased the contents of free glutamate more than 20‐fold from 8.7 ± 0.1 mg/L to 188.7 ± 1.2 mg/L. Elevated glutamate levels led to an umami taste perception in aqueous solution and taste‐enhancing properties in vegetable broth. Noteworthy, the fermentate itself exhibited an intrinsic peptidase activity. Without addition of auxiliary peptidases, mycelial enzymes caused a DH of 33.9% ± 0.7% and a free glutamate content of 99.1 ± 0.7 mg/L. For these samples, an increase in umami taste was only observed in vegetable broth, but not in water, indicating taste‐enhancing properties but low umami taste. In addition to the nutritional and health benefits of fungi, their hydrolysates are of great interest for use as a protein booster with flavor‐enhancing properties.

## Graphical abstract

Potato pulp was successfully utilized as a substrate for submerged cultivation of *Flammulina velutipes*. The fermentate was enzymatically hydrolyzed by a peptidase, and a degree of hydrolysis of 75.1% ± 1.0% was achieved. The protein hydrolysis increased the contents of free glutamate more than 20‐fold and led to an umami‐taste perception in aqueous solution and taste‐enhancing properties in vegetable broth.

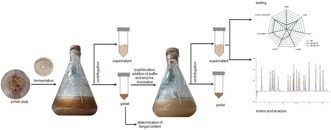

## 1 Introduction

Potato starch, which is produced in large quantities, is characterized by the formation of clear gels with a neutral taste (Grommers and van der Krogt 2009). Potato pulp, resulting as a side stream from starch production, has been used as animal feed for a long time (Friend et al. 1963). This is still the main use of potato pulp, mostly in areas close to starch mills (Wang et al. 2010). Potato pulp consists primarily of water, with starch and fiber making up most of the dry matter (Saito et al. 2006; Gao et al. 2012). Despite its high water content, potato pulp behaves more like a colloid than a liquid (Mayer 1998), which is caused by the high water binding capacity mediated by its high pectin content (Ramasamy et al. 2014). The high water binding capacity makes drying very energy intensive (Mayer 1998) and therefore limits the shelf life of potato pulp.

For this reason, fermentation is considered a more advantageous approach for the preservation of potato pulp than drying. Fermentation may be used either to reduce the water binding capacity (Du et al. 2018) or to produce lactic acid (Oda et al. 2002; Saito et al. 2007; Dagaerbieke et al. 2019). Besides, potato pulp has been tested as a substrate for some ascomycetous fungi aiming at the production of single cell protein for feed (Schügerl and Rosen 1997; Liu et al. 2013; Patelski et al. 2020).

Besides the commonly used bacteria and ascomycetes, basidiomycetes are especially promising organisms for the fermentation of potato pulp. The cultivation of mushrooms is of great interest due to their nutritional properties and the production of many nutritionally and pharmacologically active substances as well as their interesting aroma compounds and enzymes (Devi et al. 2020; Sommer et al. 2021). As an alternative to the traditional production of fruiting bodies, fungal mycelia may be cultivated in submerged liquid culture. The use of by‐products is economically interesting for mushroom cultivation as they are cheaper than synthetic media and costly disposal of the by‐products can be avoided (Fazenda et al. 2008). Fermentation can be used to enhance the sensory and nutritional value of by‐products, and a variety of such materials have been successfully fermented in submerged cultures. Some of the resulting fermentates have already been tested in food applications (Ahlborn et al. 2019; Bickel Haase et al. 2024; Sommer et al. 2023; Klis et al. 2023). The protein content can be increased by fermentation, and the protein of basidiomycetes is typically characterized by high biological values (Ahlborn et al. 2019; Bickel Haase et al. 2024).

In addition to the direct use in foods, protein hydrolysates of fermentates are considered valuable and tasteful food ingredients. Peptidases are used to enhance the digestibility of products and to produce bioactive peptides (Tavano et al. 2018). In addition, they can improve the techno‐functional properties of products and open up new applications depending on the degree of hydrolysis (DH) (Ghribi et al. 2015). Protein hydrolysates may improve the sensory profile by the production of flavor‐enhancers such as free glutamate and taste‐active peptides (Imm and Lee 1999; Poojary et al. 2017; Gao et al. 2021). The latter were identified from various hydrolyzed samples (Kong et al. 2017; Zhao et al. 2021; Yu et al. 2018). In addition, kokumi peptides may be released by protein hydrolysis (Yan et al. 2021; Moore et al. 2021). Kokumi active peptides have a low intrinsic flavor in water but a flavor‐enhancing effect in terms of continuity, mouthfeel, and thickness when added to various foods (Ueda et al. 1990; Ueda et al. 1997). Fungi have already been hydrolyzed previously, but particularly the fruiting bodies and not their submerged cultivated mycelia (Kong et al. 2019; Moore et al. 2021; Poojary et al. 2017; Gao et al. 2021).

The most commonly cultivated mushroom is *Agaricus bisporus*, followed by shiitake (*Lentinula edodes*), oyster mushrooms (*Pleurotus* spp.) and enokitake (*Flammulina filiformis*) (Valverde et al. 2015). *F. filiformis* was long thought to be conspecific with its close relative *Flammulina velutipes*, the wild enoki. Lately, DNA sequencing revealed them as two distinct species (Wang et al. 2018), but the names are still often used interchangeably. In addition to its use as an edible wild mushroom, *F. velutipes* has been described to produce interesting fatty acids as precursors for aroma compounds as well as many bioactive secondary metabolites and polysaccharides (Hammer et al. 2020; Fukushima‐Sakuno 2020; Wang and Zhang 2021).

*F. velutipes* represents a promising candidate for the fermentation of potato pulp due to its widespread use as an edible mushroom with high palatability and its bioactive constituents. The aim of the current study was thus to ferment the readily available side stream potato pulp with *F. velutipes*. Proteins of the resulting biomass were enzymatically hydrolyzed, and their sensory properties were studied.

## 2 Materials and Methods

### 2.1 Microorganism and Substrate

*Flammulina velutipes* (FVE) and *Kuehneromyces mutabilis* (KUM) were purchased from the German culture collection of microorganisms and cell cultures, DSMZ, Braunschweig, Germany (FVE: DSM 1658; KUM: DSM 1013). *Pleurotus pulmonarius* (PSP) and *Macrolepiota procera* (MPR) were provided by the Institute of Food Chemistry and Food Biotechnology of the Justus‐Liebig University, Giessen, Germany. Frozen potato pulp was obtained from Fraunhofer IVV in Freising, Germany, as a by‐product after removing potato peels, the isolation of potato starch, and the separation of potato fruit water.

### 2.2 Fermentation of Potato Pulp

For the preparation of precultures, 2 cm^2^ of a freshly grown mycelium from an agar plate was added to 200 mL of malt extract medium (20 g/L). After homogenization with an IKA ultraturrax (4000 g; 30 s), the cultures were incubated on a horizontal shaker at 150 rpm and 24°C for 7 days in the dark. The main culture medium was prepared with 20 g DM/L or 30 g DM/L potato pulp or 20 g DM/L malt extract medium in drinking water (autoclaved, 20 min, 120°C). Homogenization and incubation were then carried out as for the precultures. The biomass was harvested by pressing the fermentation broth through a cheesecloth and then lyophilized.

### 2.3 Estimation of the Fungal Content via Quantitation of Ergosterol

The fungal content was estimated by quantitation of ergosterol via the method reported by Bickel Haase et al. (2024). The ergosterol contents of cultures grown on potato pulp were compared to those of cultures grown on malt extract to estimate the fungal content of the fermentates.

### 2.4 Azocassein Assay

Peptidase activities were determined by an azocasein assay according to Kilcawley et al. (2002). Stock solutions were prepared with 5 μL enzyme and 100 mL phosphate buffer (0.17 M potassium hydrogen phosphate trihydrate, 27 mM potassium dihydrogen phosphate, pH 7.5). 160 μL of azocasein solution (10 mg azocasein, 100 mL 1 mM calcium chloride solution, 900 mL 100 mM phosphate buffer; 40°C) was added to 40 μL of enzyme buffer solution or 40 μL of culture supernatant. After addition, the preparation was homogenized on a vortex shaker for 30 s and incubated for 15 min at 40°C. Unreacted azocasein was precipitated with 300 μL of 5% trichloroacetic acid (TCA) and the solution was centrifuged at 12,000 *g* for 10 min at room temperature. One unit of enzyme activity was defined as the amount of enzyme that caused a change in absorbance of 0.01 per minute under the selected conditions. A blank was included to which 5% TCA was added prior to incubation; otherwise, the procedure was the same as for the samples. All analyses were performed in triplicate.

### 2.5 Hydrolysis of Fungal Mycelium

For optimization of the protein hydrolysis, 2 g of freeze‐dried mycelium was mixed with 100 mL of phosphate buffer (0.2 M, pH 7.5) and varying amounts of Corolase APC (AB Enzymes GmbH, Darmstadt). The incubation was performed on an incubator shaker at 200 rpm for different times and temperatures (Table 1).

**TABLE 1:** Variation of reaction parameters during hydrolysis of fungal mycelium.

Enzyme addition [mL/100 mL]	Incubation temperature [°C]	Incubation time [h]
Variation of incubation temperature		
1.00	30	20
1.00	40	20
1.00	50	20
1.00	60	20
Variation of incubation time		
1.00	40	5
1.00	40	10
1.00	40	15
1.00	40	20
Variation of enzyme addition		
0.50	40	10
1.00	40	10
2.00	40	10
3.00	40	10
4.00	40	10

The hydrolysis was subsequently carried out on a larger scale to produce samples for sensory evaluation. For this purpose, 10 g of freeze‐dried and ground mycelium was mixed with 500 mL of phosphate buffer and Corolase APC in a 1 L conical flask. The flasks were sealed with a cellulose stopper and wrapped with Parafilm. Enzymatic hydrolysis was performed at 40°C on a horizontal shaker at 200 rpm for 10 h. After incubation, the enzymatic reaction was stopped by heating at 95°C for 10 min and the samples were centrifuged (4500 *g*, 15 min, room temperature). Two blanks without enzymes were included. One blank was heat‐treated before incubation (mycelium blank; BV M) and the other after the incubation period (auto‐hydrolysis blank; BV AH). The blanks were otherwise treated in the same way as the enzyme‐treated samples.

### 2.6 Formol Titration

Formol titration was carried out according to del Navarrete Toro and García‐Carreño (2003), with minor modifications. Briefly, 25 mL of the supernatants, obtained from the blank or hydrolysis samples, was adjusted to pH 8.1 with 1 M NaOH. Then, 10 mL of formaldehyde solution (37%; pH 8.1) was added. One minute after addition, the pH was re‐titrated to 8.1 with 0.05 M sodium hydroxide standard solution (Carl Roth, Karlsruhe). To check the linearity of the formol titration, a 50 mM phenylalanine stock solution was prepared in phosphate buffer (0.2 M, pH 7.5). Solutions containing 5, 10, 15, 20, 25, and 30 mM phenylalanine were titrated (Figure S1).

### 2.7 DH

The titration results of the samples were compared with those of a total hydrolysis. Therefore, 0.25 g of lyophilised mycelium was mixed with 12 mL of 6 M HCl. After incubation for 24 h at 110°C, 12.5 mL of phosphate buffer (0.2 M; pH 7.5) and 7.5 mL of 7.5 M NaOH were added. The pH was adjusted to 8.1, and the solution was then used for formol titration. A blank without mycelium was included. The DH was calculated from the consumption of 0.05 M sodium hydroxide standard solution in the formol titration per 25 mL of the different samples (a) according to the following formula.

$$\mathrm{DH}\;\left[\%\right]=\frac{\left(a_{\text{sample}}\;\left[\mathrm{mL}\right]-a_{\text{blind value}}\;\left[\mathrm{mL}\right]\right)}{\left(a_{\text{total hydrolysis}}\left[\mathrm{mL}\right]-a_{\text{total hydrolysis},\text{blind value}}\;\left[\mathrm{mL}\right]\right)}∙100$$

### 2.8 Determination of Amino Acids and Calculation of the Biological Value

Amino acid analyses were carried out according to Ahlborn et al. (2019) for the mycelium and the supernatant as well as the remaining pellet after hydrolysis. To a sample containing ~1 mg N (determined via the method of Kjeldahl 1883 and Matissek and Fischer 2021), 2.5 mL of aqueous hydrochloric acid (6 mol/L) supplemented with 1 g/L phenol was added. The mixture was incubated at 110°C for 24 h and then cooled in an ice bath. 1.5 mL of sodium hydroxide solution (7.5 mol/L) was added and the pH was adjusted to 2.2 with sodium hydroxide solution (7.5 and 1 mol/L). For the determination of cysteine and methionine, the samples were oxidized prior to hydrolysis with 0.5 mL of the oxidation reagent (0.05 mL hydrogen peroxide (w = 30%), 0.45 mL phenolic formic acid (889 g formic acid, 111 g water, 4.73 g phenol) 1 h at room temperature) and incubated for 16 h at 4°C. Afterwards, 84 ± 1 mg of sodium metabisulphite was added before continuing with the hydrolysis and the following steps as described above. For the determination of tryptophan, the samples were hydrolyzed with 2.5 mL aqueous sodium hydroxide solution (5 mol/L with 1 g/L phenol) at 110°C for 24 h. After incubation, 1 mL of phosphoric acid (0.5 mol/L) was added and the pH was adjusted to 2.2 with hydrochloric acid (3.5 mol/L). The volume of all samples was adjusted to 20 mL with citrate buffer (11 g trisodium citrate dihydrate, 6 g citric acid, 14 mL thiodiglycol, 12 mL of a 32% (w/w) HCl and 2 g phenol ad 1 L with water, pH 2.2) in a volumetric flask, and the solution was filtered through a syringe filter (0.4 μm).

The amino acid composition was determined using an amino acid analyzer (S433, Sykam, Fürstenfeldbruck) with an LCA K13/Na column and an upstream LCA K04/Na ammonia filtration column (Sykam). The injection volume was 100 μL and a gradient of two sodium citrate buffers (0.12 mol/L, pH 3.45 and 0.20 mol/L, pH 10.85) and a regeneration solution (20 g/L sodium hydroxide, 0.2 g/L EDTA) with a flow rate of 0.45 mL/min was used for separation. The amino acids were post‐column derivatized at 130°C with ninhydrin (0.2 mol/L, pH 10.85, 0.25 mL/min) and the reaction loop was washed after each run (ethanol:isopropanol:water volume ratio 1:1:2). Identification and quantitation were performed by an external five‐point calibration (10–200 nmol/mL) of an amino acid calibration mixture (Sykam) and a prepared tryptophan solution (L‐tryptophane ≥ 99% Carl Roth, Karlsruhe).

The amino acid content, the total protein content, and the biological value were calculated as described by Ahlborn et al. (2019).

### 2.9 Determination of Free L‐Glutamic Acid

A test kit from R Biopharm (Darmstadt) was used to determine the concentration of free L‐glutamic acid in the supernatants of the hydrolysis samples according to the manufacturer's instructions.

### 2.10 Sensory Evaluation

The sensory evaluations were carried out in a test laboratory in accordance with DIN 10962 1997. The test room was odorless and noiseless. Tap water and unsalted crackers were provided for neutralization in all sensory tests. All samples submitted for sensory testing were coded with a random three‐digit numeric code. The pre‐trained panel (8 male, 12 female) consisted of 20 subjects aged between 21 and 34 years (mean 27 years). All subjects were non‐smokers. Nose clips were used for the tastings.

#### 2.10.1 Preparation of the Vegetable Broth

For the vegetable broth, 10 carrots, 10 parsnips, 6 onions, 3 stalks of celery, 6 leeks, and 6 bunches of parsley were washed and cut into cubes or rings. The vegetables were cooked for 4 min with about 120 mL of olive oil. Then, 8 L of water was added. The stock was cooked for 1 h at low heat with the lid on. The vegetables were separated from the liquid with a sieve, and 5 g/L of NaCl was added.

#### 2.10.2 Triangle Test of Difference

Hydrolyzed samples and blind values were analyzed by a triangle test for difference. Two vegetable broth samples with 8 mL vegetable broth and 2 mL phosphate buffer (0.2 M, pH 7.5) and one outlying sample (8 mL vegetable broth, 2 mL hydrolyzed sample suspension) were provided. All samples were heated to 60°C (for about 1 h) in an oven (HT6B60F0, Siemens AG, Germany) shortly before the tasting. The panelists were asked to identify the outlying sample. An *α*‐risk of 0.05 was defined for the analysis of the triangle test.

#### 2.10.3 Descriptive Test

The vegetable broth and the hydrolyzed samples in vegetable broth were used for a descriptive test. In addition, the hydrolyzed samples and blanks were described after they were diluted 1:5 with drinking water and heated to 60°C in an oven.

The samples were compared to standard solutions in a descriptive test. Caffeine was used in a concentration of 0.125 g/L as a reference solution for bitter, sodium chloride in a concentration of 0.65 g/L for salty, and citric acid monohydrate with 0.25 g/L for acidic. A sucrose solution (4.00 g/L) was used as a sweet reference and sodium L‐glutamate monohydrate (0.36 g/L) for umami. The reference substances were dissolved in drinking water. The samples were also evaluated for the attributes “full‐bodied” and “overall impression”. The intensity of the standards was set at 5 on a scale up to 10.

### 2.11 Statistical Analysis

The means and standard deviations are shown and were determined with Excel. Statistical analyses were done with GraphPad Prism 9.5.1 using an unpaired t test or an analysis of variance (ANOVA). A significance level of *p* < 0.05 was selected. Parameters for the triangle sensory test are mentioned in the respective chapter.

## 3 Results and Discussion

### 3.1 Production of Fungal Mycelium

As the potato pulp used in the fermentation medium as a sole source of carbon and nitrogen is not completely soluble, the biomass resulting from the fungal fermentation may contain residual undigested substrate. The fungal content of the fermentates was thus determined using ergosterol as a biomarker. The fungal content, the biomass, and the protein content of potato pulp fermented with four basidiomycetes were analyzed to determine which fungus optimally converts the substrate to fungal biomass. These candidates were identified in a prescreening on agar plates and in submerged culture (data not shown). Of the four fermentates, the one with *F. velutipes* showed the highest fungal content of 96% ± 2% and was chosen for further experiments (Figure S2). To achieve higher biomasses, the substrate concentration was increased from 20 g/L (as used in the screening) to 30 g/L. For the *F. velutipes* fermentate, a high fungal content of 83% ± 3% was still achieved with the increased substrate concentration. This fungal content was significantly higher than the up to 54% fungal content of cocoa pod shells fermented with *Pleurotus salmoneo‐stramineus* (Bickel Haase et al. 2024) and similar to the 89% fungal content when *Pleurotus sapidus* was grown on apple pomace using a fed‐batch approach (Ahlborn et al. 2019).

### 3.2 Enzymatic Hydrolysis of Fungal Mycelium

Once the biomass was produced and lyophilized, it was suspended in buffer and treated with a commercial peptidase. The efficiency of enzymatic hydrolysis depends on various factors, such as the specificity of the applied enzyme, the type of substrate, pH, temperature, and incubation time (Arteaga et al. 2020; Guan et al. 2018; Noman et al. 2018). To optimize enzymatic hydrolysis, different parameters were varied, and the resulting mixtures were compared by means of formol titration.

**FIGURE 1:**
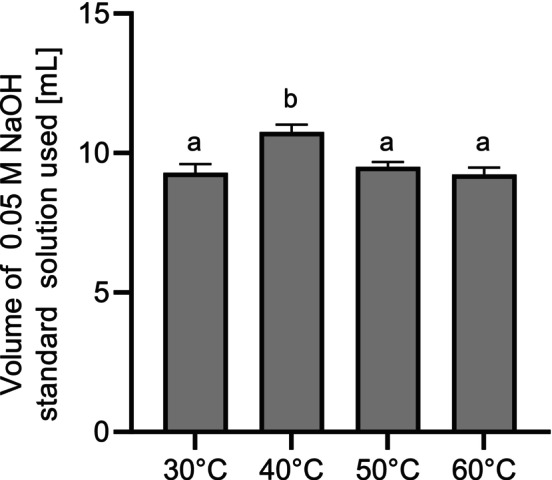
Volume of 0.05 M NaOH standard solution used [mL] for formol titration of ME1 after the incubation at different temperatures [°C] (*n* = 3). Incubation time: 20 h. ME1: Enzymatically hydrolyzed mycelium with 1 mL of enzyme. Means with no letter in common indicate significant differences (*p* < 0.05).

**FIGURE 2:**
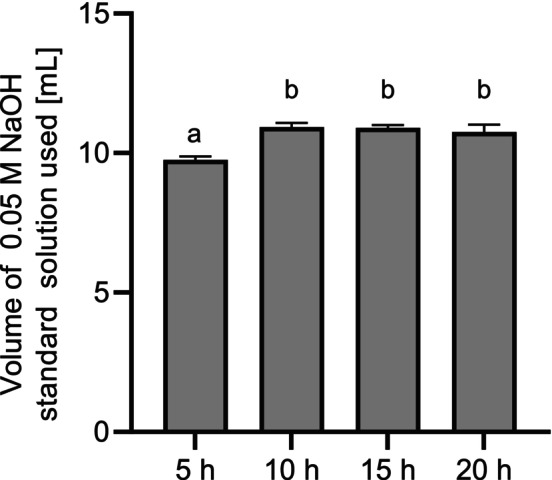
Volume of 0.05 M NaOH standard solution used [mL] for formol titration of ME1 after different incubation times [h] (*n* = 3). Temperature: 40°C; enzyme addition: 1 mL enzyme to 100 mL buffer and 2 g mycelium. ME1: Enzymatically hydrolyzed mycelium with 1 mL of enzyme. Means with no letters in common indicate significant differences (*p* < 0.05).

The variation of the incubation temperature between 30°C and 60°C (Figure 1) as well as the incubation time from 5 to 20 h (Figure 2) was tested with an enzyme addition of 1 mL to 100 mL sample suspension (ME1).

**FIGURE 3:**
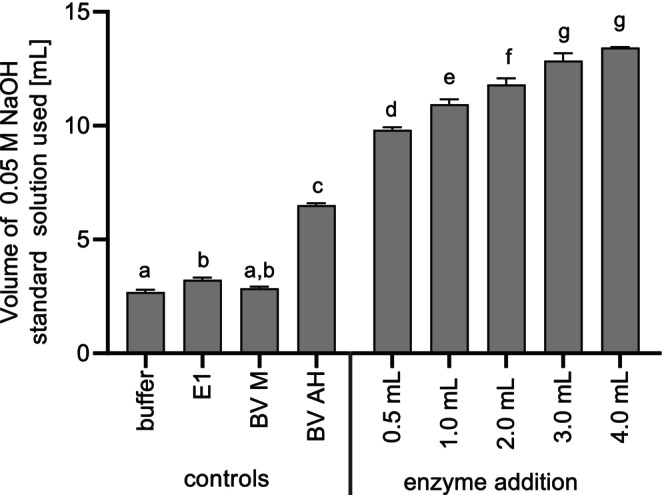
Volume of 0.05 M NaOH standard solution used [mL] for formol titration of controls (*n* = 3) and samples with different enzyme addition to 100 mL sample suspension [mL] (*n* = 2). Temperature: 40°C; incubation time: 10 h. E1: 1 mL enzyme in buffer; BV M: Heat‐inactivated mycelium before incubation; BV AH: Incubated mycelium without enzyme. Means with no letter in common indicate significant differences (*p* < 0.05).

Formol titration revealed an optimum incubation temperature of 40°C. The enzyme activity of Corolase APC was determined at different temperatures using an azocasein assay, with an optimum at 65°C (Figure S3). This result differs from the use of the enzyme to hydrolyze the fungal mycelium. The incubation period of up to 20 h for hydrolysis was much longer than the 15 min for the enzyme assay. This has probably affected the stability of the enzyme due to denaturation. In addition, hydrolysis after the addition of the commercial peptidase results from a combination of endogenous fungal peptidases and the added enzyme, which could exhibit additive or synergistic effects. Synergistic effects have been observed, for example, in the combination of different peptidases used for the hydrolysis of soy protein isolates (De Castro and Sato 2015). As the preferred growth temperature of *F. velutipes* is usually around 25°C (Kong et al. 2004) and fungal peptidases might be more active at optimal growth conditions, temperatures below 65°C during the hydrolysis process resulted in higher hydrolysis rates even though the Corolase APC temperature was not at the optimum level. A reaction temperature of 40°C was therefore used for further experiments. The amount of required NaOH standard solution increased significantly from 5 to 10 h (Figure 2). After an incubation time of 10 h, no further increase was observed. The further hydrolysis experiments were thus performed with an incubation time of 10 h. The amount of enzyme was varied between 0.5 and 4.0 mL per 100 mL of sample suspension (Figure 3).

To test whether the addition of enzyme affected the assay, a control was included with the addition of 1 mL of enzyme to 100 mL of buffer (E1) (Figure 3). A control of the mycelium without enzyme addition was also tested using an inactivation step prior to the incubation period (BV M). The consumption of NaOH was the same for both controls, indicating that neither the biomass itself nor the enzyme affected the assay. As there was no difference between the buffer control and BV M, the inactivation step at 90°C for 10 min was sufficient to inactivate the fungal peptidases.

To test the proteolytic activity of the mycelium, no enzyme was added, and the preparation was incubated (BV AH). A clear difference was found compared to the other controls. This can be explained by the activity of fungal peptidases (Figure 3). Basidiomycetes are able to express a wide variety of different hydrolases, including peptidases (Sabotič et al. 2007). The supernatant from submerged cultures of *F*. *velutipes* is reported to have high proteolytic activity compared to other basidiomycetes (Grimrath et al. 2011).

The proteolytic activity of the fungal peptidases was further characterized by the azocasein assay of the supernatant after fermentation of potato pulp with *F. velutipes*. The activity of the supernatant was 118.9 ± 2.3 U/mL and thereby slightly lower than the extracellular peptidase activity of *F*. *velutipes* when cultured on gluten with a maximum peptidase activity of 269.8 U/mL, but still high in comparison to peptidase activities of other basidiomycota (Grimrath et al. 2011). The activities of the supernatant were in a comparable range to the activities of commercial enzymes such as flavourzyme (153 U/mL) or papain (139 U/mL) (Waglay and Karboune 2016).

Peptidases from *F. veluitpes* have already been identified (Schulz et al. 2018; Iketani et al. 2013). However, the molecular characterization of the peptidases in the supernatant and the search for other peptidases may be of interest. In addition to the identification and targeted use of additional peptidases, the use of the fermentation supernatant as an enzyme mixture is also conceivable. This principle has already been demonstrated for *Aspergillus oryzea*, where peanut protein was hydrolyzed with lyophilized fermentation supernatant (Su et al. 2011; Zhang et al. 2019) or for the use of supernatant and whole fermentation broth of *Trichoderma reesei* and *Trichoderma atroviride* for the hydrolysis of pretreated spruce (Kovács et al. 2009).

### 3.3 Total Hydrolysis and Calculation of the DH

The fungal mycelium was freeze‐dried and ground prior to enzymatic treatment. The DH was determined to be 33.9% ± 0.7% for BV AH and 75.1% ± 1.0% for ME1. When 4 mL of enzyme was added (ME4) to 100 mL of sample suspension, the volume of NaOH required was almost the same as that for chemical total hydrolysis, resulting in a DH of 98.1% ± 0.2%. The influence of different drying methods on the amount of free amino acids in *F. velutipes* fruiting bodies has been reported previously, with the highest amounts found after lyophilization (Wang et al. 2023). However, as there was no difference between the buffer control and BV M, it can be excluded that the pre‐treatment of the mycelium had an effect on the DH. Various plant and animal‐derived proteins have been hydrolyzed with a varying set of enzymes. By treating an okara protein concentrate with a mixture of endo‐ and exopeptidases, a DH of 22% and an increase in antioxidant capacity were achieved (Pereira et al. 2019). The hydrolysis of casein and whey protein resulted in a DH of up to 24%, and the enzyme treatment had an effect on the properties of the emulsions (van der Ven et al. 2001). A DH of up to 9.2% has been achieved with various enzymes hydrolyzing pea protein. A Corolase was also included (Corolase 7089) and showed a DH of only 4.7% (Arteaga et al. 2020). The DH of soya protein with Corolase PP treatment for 4 h under various conditions, such as variation of pressure, reached a maximum of 30.6% (Guan et al. 2018). The highest DH obtained by hydrolysis of pretreated defatted soya flakes with various proteases was 49.3% (Chae et al. 1998). A higher DH of 68% was achieved for the hydrolysis of soluble fish concentrate (Nilsang et al. 2005). Potato juice, as another by‐product of potato starch production, contains the protein fraction after starch isolation (Kot et al. 2020). Treatment of potato juice with various enzymes and conditions resulted in a maximum DH of 44% (Kamnerdpetch et al. 2007) and enzymatic hydrolysis of potato juice increased the antioxidant and in vitro cytotoxic activity (Kowalczewski et al. 2021). When various commercial enzymes and conditions were tested for the hydrolysis of isolated potato protein, DHs of up to 100% were achieved (Waglay and Karboune 2016). Fruiting bodies of fungi have also been hydrolyzed previously with a maximum DH of 28% (Poojary et al. 2017), 37% (Gao et al. 2021) or 67% (Ang and Ismail–Fitry 2019), depending on the enzyme and fungus combination. The DH of most previous studies was below or in the range of BV AH, emphasizing the potential of *F. velutipes* peptidases.

### 3.4 Amino Acid Analysis of Potato Pulp and the Fermentates

Potato pulp, the solid by‐product of potato starch production, has not yet been treated with proteases to produce protein hydrolysates. One reason for this may be that it contains only about 5.3 ± 0.4 g/100 g DM protein. By fermenting the potato pulp with *F*. *velutipes* (PP FVE), the protein content of the biomass was significantly increased to 13.9 ± 0.1 g/100 g DM. The fermentation of side streams with basidiomycetes may offer an effective method to increase the protein content of side streams, as also reported for other fungus/substrate combinations (Ahlborn et al. 2019; Bickel Haase et al. 2024) and may be of interest for many more industrial side streams. The submerged cultivation of *F. velutipes* on standard media resulted in protein contents of 17.5 g/100 g DM (Kozhemyakina et al. 2010) and 18.7 g/100 g DM (Hassan et al. 2012). The protein content of PP FVE could be further increased by adding external nitrogen sources, as done in the fermentation of other by‐products (Bickel Haase et al. 2024), but due to the very good fungal growth on potato pulp (fungal content > 80%), fermentation without an external nitrogen source seems more attractive from an economic point of view.

**FIGURE 4:**
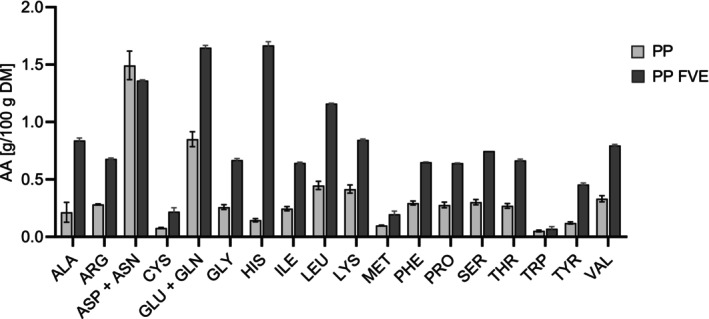
Amino acid composition of the freeze‐dried potato pulp (PP) und potato pulp fermented with *Flammulina velutipes* (PP FVE) (*n* = 2).

The amino acid analysis showed that all essential amino acids were present in PP FVE, and the fermentation resulted in a significant increase in most amino acids except for aspartate/asparagine and tryptophan (Figure 4). The largest increase was observed for histidine, which was the most abundant amino acid after fermentation, together with the sum of glutamine and glutamate.

The biological value of the PP FVE protein was 86 ± 1, which is high compared to plant protein sources and comparable to or higher than those of protein sources of animal origin. The biological value of beef is 80, of milk 91, of soy 74, and of wheat 64 (Hoffman and Falvo 2004). Many fungi are able to produce protein with high biological value, which indicates a high nutritional value for humans. The protein of *P. sapidus* grown on apple pomace and the protein from cocoa pod husks fermented with *P. salmoneo‐stramineus* had the same biological value of 86 (Ahlborn et al. 2019; Bickel Haase et al. 2024). When comparing the protein of fruiting bodies of three different *Pleurotus* strains, the highest biological value was 88 (Del Toro et al. 2006).

Overall, potato pulp was shown to be a highly suitable substrate for the submerged cultivation of *F. velutipes*, and high fungal contents of the fermentates as well as an increase in the protein contents were achieved.

### 3.5 Amino Acid Analysis after Hydrolysis

**FIGURE 5:**
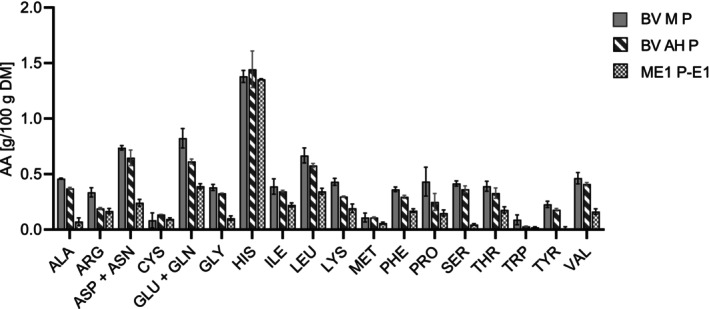
Amino acid composition of the freeze‐dried hydrolysate pellet samples (P) (*n* = 2). BV M: Heat inactivated mycelium before incubation; BV AH: Incubated mycelium without enzyme; ME1: Enzymatically hydrolyzed mycelium with 1 mL of enzyme; E1: 1 mL enzyme in buffer.

**FIGURE 6:**
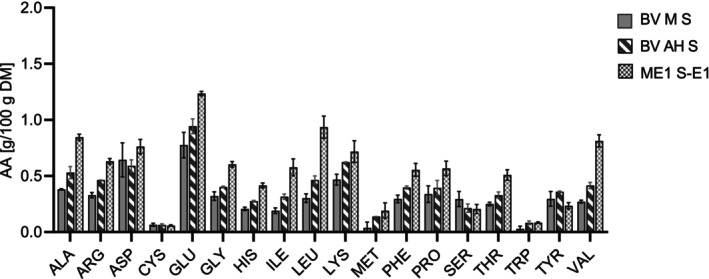
Amino acid composition of the freeze‐dried hydrolysate supernatant samples (S) (*n* = 2). BV M: Heat‐inactivated mycelium before incubation; BV AH: Incubated mycelium without enzyme; ME1: Enzymatically hydrolyzed mycelium with 1 mL of enzyme; E1: 1 mL enzyme in buffer.

For the hydrolyzed samples and the controls, the sum of amino acids (Table 2) and the amino acid distribution were analyzed for the pellet (Figure 5) and the supernatant (Figure 6). The content of amino acids in the supernatant increased with the DH, whereas it decreased in the pellet. This additionally reflects the increasing DH. The contents of valine, leucine, and alanine were comparatively high. The sum of glutamate and glutamine made up the largest share of amino acids in the supernatant after hydrolysis. This is particularly interesting due to the role of glutamate for the umami and overall taste (Ikeda 1909).

**TABLE 2:** Sum of amino acids (AA_sum_) in PP FVE and in the various fractions after hydrolysis and centrifugation (*n* = 2).

	PP FVE	BV M	BV AH	ME1‐E1
P AA_sum_ [g/100 g DM]	—	8.2 ± 0.1	7.0 ± 0.2	4.0 ± 0.2
S AA_sum_ [g/100 g DM]	—	5.5 ± 0.3	7.0 ± 0.2	9.9 ± 0.2
T AA_sum_ [g/100 g DM]	13.9 ± 0.1	13.7 ± 0.2	13.9 ± 0.1	13.9 ± 0.1

Abbreviations: BV AH: incubated mycelium without enzyme; BV M: heat‐inactivated mycelium before incubation; E1: 1 mL enzyme in buffer; ME1: enzymatically hydrolyzed mycelium with 1 mL of enzyme; P: pellet; PP FVE: potato pulp fermented with *Flammulina velutipes*; S: supernatant; T: total.

### 3.6 Quantitation of Free L‐Glutamic Acid

Amino acids impart different sensory impressions. While leucine, phenylalanine, tryptophan, tyrosine, and glycine exhibit a bitter taste in aqueous solution, alanine and glycine show a sweet taste, and proline and methionine have a slightly sweet taste. Apart from the mild sweet taste, methionine also tastes sulfurous and meaty, and cysteine tastes sulfurous (Solms 1969; Schiffman et al. 1981). Glutamate plays an important role in flavor and was identified as a key component of umami flavor already in 1909 (Ikeda 1909). It is not possible to distinguish between free amino acids and those bound in peptides or proteins or between glutamate and glutamine after total hydrolysis. Therefore, the content of free L‐glutamic acid was determined in the supernatant of the hydrolysis samples, the blank values without enzyme addition (BV M; BW AH) and the vegetable broth (VB), which was used for the sensory tests (Table 3).

**TABLE 3:** Concentration of free L‐glutamic acid of hydrolyzed samples, controls, and vegetable broth [mg/L and mg/g] (*n* = 3).

	BV M	Vegetable broth	BV AH	ME1	ME4
L‐glutamic acid	8.7 ± 0.1 mg/L (0.56 ± 0.01 mg/g)	52.1 ± 1.6 mg/L	99.1 ± 0.7 mg/L (6.23 ± 0.04 mg/g)	188.7 ± 1.2 mg/L (11.31 ± 0.71 mg/g)	214.9 ± 3.9 mg/L (10.69 ± 0.20 mg/g)

Abbreviations: BV AH: Incubated mycelium without enzyme; BV M: Heat‐inactivated mycelium before incubation; ME1: Enzymatically hydrolyzed mycelium with 1 mL of enzyme; ME4: Enzymatically hydrolyzed mycelium with 4 mL of enzyme.

The concentration of free glutamate in the supernatant was significantly increased with increasing DH. The free glutamate content of BV AH was almost twice that of the VB sample. After enzyme addition, the free glutamate content increased further, with the highest content occurring when 4 mL instead of 1 mL of enzyme was added. For comparison with the results obtained after total hydrolysis and various literature values, the glutamate content was referred to the dry mass, taking the residual moisture and the phosphate content of the buffer into account. Due to the higher mass contained in the supernatant of ME4, the glutamate content in relation to the dry mass did not change further between the addition of 1 and 4 mL of enzyme. Comparing the free glutamate in the supernatant with the total glutamine and glutamate in the supernatant, the free glutamate in BV M was about 4%, in BV AH about 40%, and in ME1 about 67% of the total content of glutamine and glutamate. These values were in good agreement with the DH of the corresponding samples, indicating glutamate to make up a larger proportion of the total amount of glutamate and glutamine than glutamine. This could be due to a higher occurrence of glutamate than glutamine in the fungal protein or to an endogenous glutaminase activity. Glutaminases have been detected in several fungi (Amobonye et al. 2022), and an asparaginase with additional glutaminase activity has been identified in *Flammulina velutipes* (Eisele et al. 2011). Because of the important role of glutamate for the umami taste and the overall flavor, glutaminases are used in soy sauce production (Wakayama et al. 2005). The observation that glutamate accounts for the majority of the glutamate/glutamine content indicates that the fungal hydrolysates do not require the additional use of glutaminases.

The contents of free glutamate of fruiting bodies of *F. velutipes* were determined in different studies. In two of them, levels of 31.54 mg/g (Kim et al. 2009) and 29.98 mg/g (Beluhan and Ranogajec 2011) were found. In other analyses, the levels of free glutamate ranged from 0.745–1.679 mg/g (Donglu et al. 2017), from 2.1–4.1 mg/g (Wang et al. 2023) and from 1.54–6.82 mg/g (Yang et al. 2001). Enzymatic hydrolysis of the fruiting bodies of various fungi increased the free glutamate content from 2.6 mg/g TM to 7.1 mg/g (shiitake, lowest values) and from 14.0 mg/g to 19.6 mg/g (white champignon, highest values) (Poojary et al. 2017). The level of glutamate in fungi depends on several factors, such as the fungal strain, the time of harvest of the fruiting bodies, and further processing and storage (Zhang et al. 2013). Nevertheless, our results are in good agreement with other described values. Overall, the free glutamate content was significantly increased by enzymatic hydrolysis in ME1, also compared to the BV AH sample.

### 3.7 Triangle Test with Descriptive Tests

The hydrolysate samples and controls were differentiated significantly in a triangle test when compared to VB. In the descriptive test, VB was described against each aberrant sample (hydrolysate samples and controls), which is why five separate evaluations of VB were conducted. No significant differences between the descriptions of VB were found, indicating a good test design with reproducible results.

**FIGURE 7:**
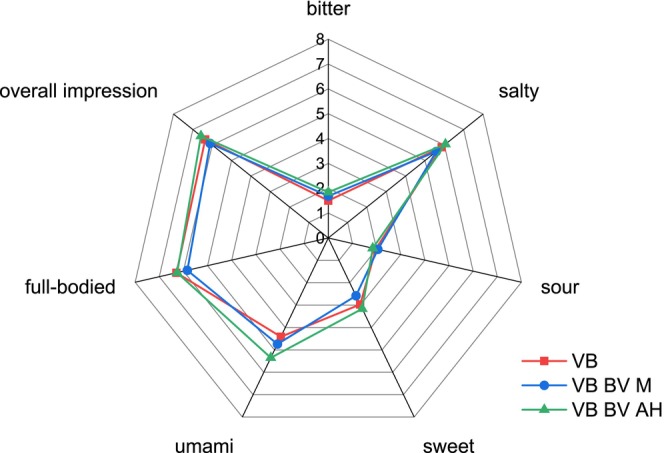
Descriptive test in vegetable broth (VB) of BV M and BV AH (*n* = 19). BV M: Heat‐inactivated mycelium before incubation; BV AH: Incubated mycelium without enzyme.

**FIGURE 8:**
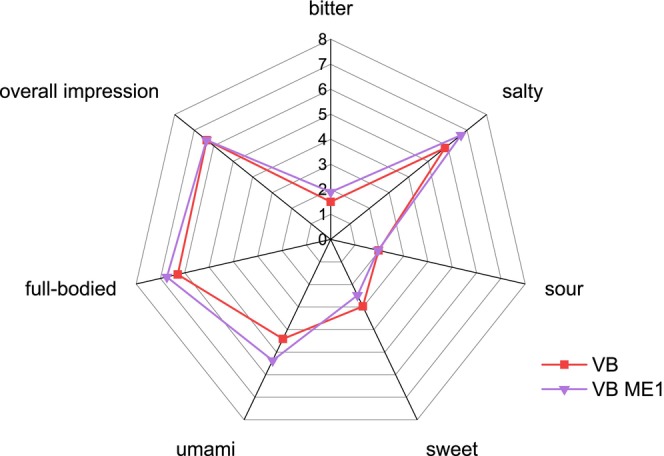
Descriptive test in vegetable broth (VB) of ME1 (*n* = 19). ME1: Enzymatically hydrolyzed mycelium with 1 mL of enzyme.

Although BV M was significantly recognized as a deviating sample in VB, no significant differences were found between the descriptive evaluation of VB and BV M (Figure 7). BV AH (Figure 7) and ME1 (Figure 8) in VB had higher ratings for the attribute umami (both 5.4) than the VB (4.4). The other attribute ratings for BV AH and ME1 were similar to those for VB.

**FIGURE 9:**
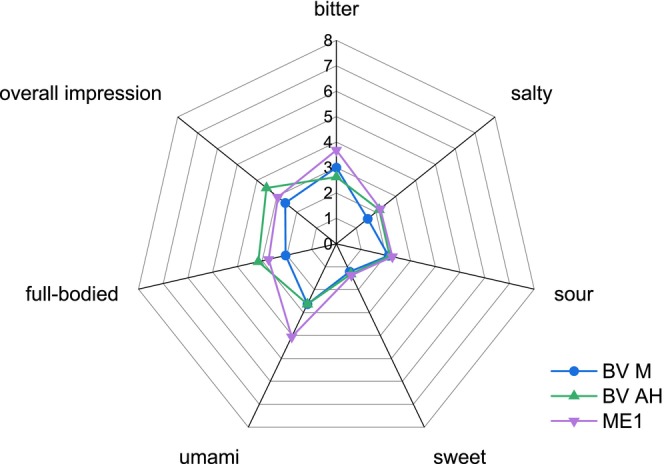
Descriptive test in water of BV M; BV AH; and ME1 (*n* = 19). BV M: Heat‐inactivated mycelium before incubation; BV AH: Incubated mycelium without enzyme; ME1: Enzymatically hydrolyzed mycelium with 1 mL of enzyme.

**FIGURE 10:**
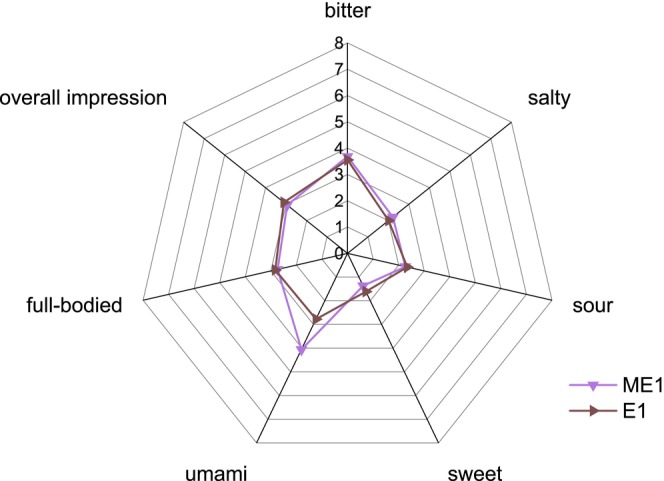
Descriptive test in water ME1 and E1 (*n* = 19). ME1: Enzymatically hydrolyzed mycelium with 1 mL of enzyme; E1: 1 mL enzyme in buffer.

In addition to the descriptive test in VB, a descriptive test was carried out by diluting the samples with water. An increase in the umami rating of ME1 was observed compared to BV M (Figure 9) and to E1 (Figure 10). In contrast to the ratings in VB, the umami ratings in water for BV M and BV AH were similar, but BV AH was perceived to be more full‐bodied than BV M.

**FIGURE 11:**
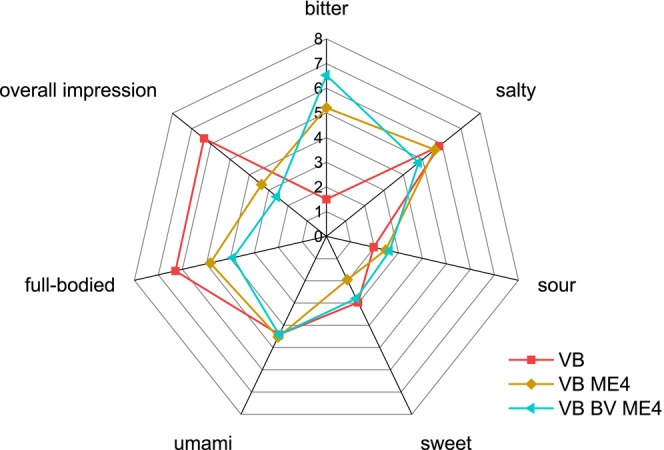
Descriptive test in vegetable broth (VB) of ME4 and BV ME4 (*n* = 19). ME1: Enzymatically hydrolyzed mycelium with 4 mL of enzyme; BV ME4: Heat‐inactivated mycelium before incubation with 4 mL of enzyme.

**FIGURE 12:**
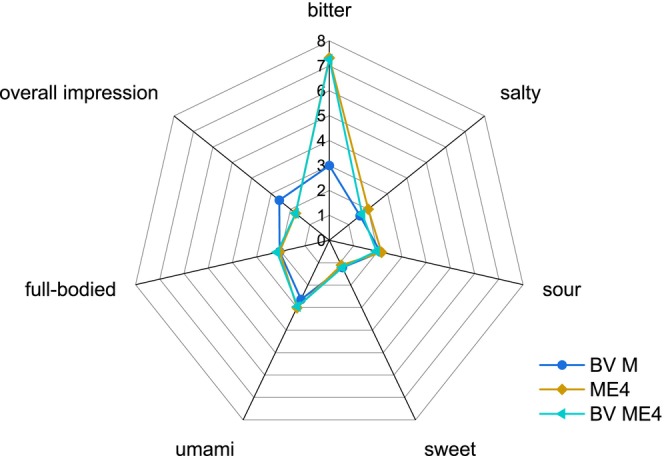
Descriptive test in water BV M, ME4, and BV ME4 (*n* = 19). ME4: Enzymatically hydrolyzed mycelium with 4 mL of enzyme; BV ME4: Heat‐inactivated mycelium before incubation with 4 mL of enzyme.

When more enzyme was used for hydrolysis, the rating for bitterness increased significantly in VB and in water (Figures 11 and 12). The ratings for overall impression and full‐bodied decreased in VB (Figure 11) as well as the rating for overall impression in water (Figure 12). The high bitter ratings were also observed when the BV ME4 sample was tasted in VB and in water, indicating that the bitter taste is partially due to the enzyme itself. Bitterness can be a problem with hydrolysates in general. It depends on the used enzymes due to their own taste or the release of bitter amino acids or peptides (Fávaro‐Trindade et al. 2010; Su et al. 2011; Spellman et al. 2009). There are several methods for reducing bitterness of hydrolysates, such as deamidation using various processes (He et al. 2019; Liu et al. 2017). Bitterness can also be a problem for the application of fungi in food products. In a comparison of different fungal fruiting bodies, all showed relatively high values for bitterness in an electric tongue test (Phat et al.2016). The fruiting bodies of *F. velutipes* exhibited the highest value for umami and a low value for sour in this analysis (Phat et al. 2016). When dried shiitake extract was used as a flavor enhancer in meat formulations, a significantly higher bitter taste was perceived (Dermiki et al. 2013) and during the hydrolysis of *Morchella sextelata* fruiting bodies, the bitter taste was increased depending on the enzyme used (Gao et al. 2021). The E1 control in water showed no increase in bitterness compared to BV M, indicating no effect of an enzyme addition at this concentration. The fact that the mycelium samples themselves (BV M; BV AH) as well as ME1 did not show an increase in bitterness in water or VB is favorable for the further use of the mycelium or hydrolysates. Since the umami flavor was increased when ME1 or BV AH were added to VB without reducing the overall score, their implementation in other food systems seems promising.

Interestingly, BV AH and ME1 showed an increase in umami perception when added to vegetable broth, but unlike ME1, BV AH did not show an increase in umami in water. This indicates an umami enhancing effect without an inherent umami taste of BV AH. The umami‐enhancing effect could be due to umami‐enhancing peptides, some of which have been previously identified in fruiting bodies of fungi (Kong et al. 2019; Chen et al. 2022; Xu et al. 2019). The increase in the rating for the full‐bodied taste of BV AH compared to BV M in water could be additionally an indication of the presence of kokumi active substances. The notion of kokumi can be influenced by the presence of glutamate as it might facilitate the binding of existing kokumi substances to kokumi receptors (Yamamoto and Inui‐Yamamoto 2023).

The increased umami taste in the water of ME1 could be explained by the free glutamate content associated with the higher DH in comparison to BV AH (Table 3). In addition, peptides smaller than 3 kDa were highly correlated with umami properties (Gao et al. 2021) indicating that a higher DH increases the umami perception. To further enhance the umami effect, an additional option would be to use BV AH and ME1 in a following Maillard reaction (Zhang et al. 2019; Yan et al. 2021; Yu et al. 2018). Experiments have also shown that small peptides of 1–3 kDa have a greater effect after the Maillard reaction not only on umami enhancement but also on the saltiness and kokumi enhancing effect (Yu et al. 2018; Yan et al. 2021), making ME1 a good candidate for use due to its high DH.

## Author Contributions

**Katharina Happel:** conceptualization; supervision; investigation; methodology; data analysis; writing – original draft. **Lea Zeller:** investigation; methodology; data analysis; writing – review and editing. **Andreas Klaus Hammer:** conceptualization, supervision, investigation; methodology; writing – review and editing. **Holger Zorn:** funding acquisition; project administration; resources; supervision; writing – review and editing.

## Conflicts of Interest

The authors declare no conflicts of interest.

## Supporting information


Data S1.

## Data Availability

The data that support the findings of this study are available on request from the corresponding author.
