# Supplementary material for: Umami Enhancing Properties of Enzymatically Hydrolyzed Mycelium of Flammulina velutipes Cultured on Potato Pulp
Source: Food Sci Nutr. 2025 Mar 31;13(4):e70128. doi: 10.1002/fsn3.70128 (PMC11958610; doi:10.1002/fsn3.70128)
Supplement: Supplementary file 1 — Data S1. [file FSN3-13-e70128-s001.docx]

Umami enhancing properties of enzymatically hydrolyzed mycelium of *Flammulina velutipes* cultured on potato pulp

Enzymatic hydrolysis *of Flammulina velutipes* mycelium

Katharina Happel^1^; Lea Zeller^1^; Andreas Klaus Hammer^1^; Holger Zorn^1,2^

1: Fraunhofer Institute for Molecular Biology and Applied Ecology IME, Giessen, Germany

2: Institute of Food Chemistry and Food Biotechnology, Justus- Liebig University, Giessen, Germany

Figure 1: Verification of the linearity of formol titration with phenylalanine solutions of different concentrations [mM] (*n*=3). Error bars depict standard deviation.

Figure 2: Fungal content [%], dry matter [g/L] and crude protein content [g/100 g DM] after cultivation potato pulp. MPR: Macrolepiota procera; KUM: Kuehneromyces mutabilis; FVE: Flammulina velutipes; PSP: Pleurotus pulmonarius (n=2).

Figure 3: Enzyme activity [U/mL] of Corolase APC (1.25 µL/100 mL) at pH 7.5, depending on temperature [°C] (*n*=3). Error bars depict standard deviation.
